# Severe acute radiation syndrome: treatment of a lethally ^60^Co-source irradiated accident victim in China with HLA-mismatched peripheral blood stem cell transplantation and mesenchymal stem cells

**DOI:** 10.1093/jrr/rrt102

**Published:** 2013-08-26

**Authors:** Mei Guo, Zheng Dong, Jianhui Qiao, Changlin Yu, Qiyun Sun, Kaixun Hu, Guangxian Liu, Li Wei, Bo Yao, Qiuhong Man, Xuedong Sun, Zhiqing Liu, Zhiwu Song, Chengze Yu, Ying Chen, Qingliang Luo, Sugang Liu, Hui-Sheng Ai

**Affiliations:** 1 Department of Hematology and Transplantation, Affiliated Hospital of the Academy of Military Medical Sciences, 8 Dongdajie, Beijing 100071, China; 2 Department of Radiation, Institute Radiation of Medical, 27 Taipinglu, Beijing 100039, China

**Keywords:** acute radiation syndrome, HLA-mismatched stem cell transplantation, mesenchymal stem cell

## Abstract

This is a case report of a 32-year-old man exposed to a total body dose of 14.5 Gy γ-radiation in a lethal ^60^Co-source irradiation accident in 2008 in China. Frequent nausea, vomiting and marked neutropenia and lymphopenia were observed from 30 min to 45 h after exposure. HLA-mismatched peripheral blood stem cell transplantation combined with infusion of mesenchymal stem cells was used at Day 7. Rapid hematopoietic recovery, stable donor engraftment and healing of radioactive skin ulceration were achieved during Days 18–36. The patient finally developed intestinal obstruction and died of multi-organ failure on Day 62, although intestinal obstruction was successfully released by emergency bowel resection.

## INTRODUCTION

Acute radiation syndrome (ARS) following an accidental nuclear exposure often induces severe bone marrow aplasia, gastrointestinal syndrome, skin burns, lung injury and/or central nervous system failure. Although allogeneic stem cell transplantation (alloSCT) and hematopoietic cell cytokines such as granulocyte colony-stimulating factor (G-CSF) are used, most patients with severe ARS die of bone-marrow failure or multi-organ failure [1–6]. The therapeutic potential of bone-marrow-derived mesenchymal stem cells (MSCs) has been well documented through animal model studies and clinical research, e.g. to hasten hematopoietic recovery and promote tissue repair following radiation injury [7–10]. However, the use of MSCs combined with alloSCT for the treatment of ARS in humans has not yet been reported. This article records the outcome for one patient who was exposed to a ^60^Co source and received 14.5 Gy irradiation in an accident in Taiyuan, China in 2008. The patient was treated with an infusion of MSCs combined with HLA-mismatched peripheral blood stem cell transplantation, and achieved speedy hematopoietic recovery.

## MATERIALS AND METHODS

The radiation accident occurred at about 13:20 on 11 April 2008 at a plant for irradiation of traditional Chinese medicine in Taiyuan, Shanxi Province, China. The victim was a 32-year-old man. There were four workers handling the Chinese medicines. At 13:36, one of them, as described in this report, saw a ‘blue light’ and found that the radiation source (cobalt source, source intensity of 18 000 Curie) was still in working position, rather than retracted to a safe location. The distance between the workers and the radiation source was 80–150 cm. They evacuated immediately to a safe place away from the scene. Frequent vomiting, facial flushing and fever (temperature 38.9°C) were observed 30 min after exposure for the victim, who was transferred to our hospital 14 h after exposure. On admission, he presented with diffuse skin erythema on hands, face and trunk, and his parotid glands were slightly swollen. At 2 h after exposure, his white blood cell (WBC) count was 14.2 × 10^9^/l. At 45 h after exposure, his lymphocyte count had decreased to 0, bilirubin was 29.9 μmol/l, aminotransferase was 46μ/l, and urea was 10.4 μmol/l. Treatments to improve hematologic recovery and prevent infections included administration of granulocyte colony-stimulating factor (G-CSF), selective gastrointestinal tract sterilization, prophylactic antibacterial and antifungal reagents, and reverse isolation.

On Day 4 after exposure, the patient developed diarrhea (60 ml per day) and signs of systemic inflammatory response syndrome such as thoracodynia, difficulty breathing, hypopiesia (blood pressure 84–56 mmHg) and hypoxemia (88% oxygen saturation). On Day 7 after exposure, the patient's general condition was improved, and hypopiesia and hypoxemia disappeared after treatment with methylprednisolone, oxygen and furosemide. However, his WBC count decreased to 0.1 × 10^9^/l, and bone marrow taps showed hypocellular marrow with decreased erythroid, myeloid and megakaryocytic lineages. Bone marrow examination suggested that there was little hope for autologous hematopoietic reconstitution and the same results were obtained by dosimetry. Biological dosimetry estimated from the decline rate of WBC and lymphocytes was > 10Gy, Cytogenetic studies indicated 14.5 (11.4–17.9) Gy γ-radiation, depending on the method of γ-dose estimation (Table 1) [11]. However, there was no matched related donor available. In our hospital the patient received HLA-A, B, C, DR and DQ mismatched haplo-identical peripheral blood transplantation from his older brother, combined with mesenchymal stem cells. Fludarabine (100 mg) was administered as an emergency conditioning on Day 7. Mononuclear cells and CD34+ cells were infused (11.56 × 10^8^/kg and 8.54 × 10^6^/kg, respectively) on Day 8 after exposure. Cyclosporine A (CSA, 1.5 mg/kg, transvenously), mycophenolate mofetil (MMF, 30 mg/kg) and CD25 monoclonal antibody (20 mg) were used for graft-versus-host disease (GVHD) prophylaxis [12].

**Table 1.** Dosimetry of the victim

Method	Dosage (Gy)
Decline rate of WBC and lymphocytes	>10
Dic + Rc	14.5 (11.4–17.9) 15.3 (13.7–16.9)^a^
CBMN + NDI	10–20
PCC-R	12.4 (9.5–15.3)
Physics method	9.8 (6.9–14.7)^b^

Dic + Rc = dicentris plus centric ring, CBMN + NDI = cytokinesis-block micronuclei plus nuclear division index, PCC-R = premature chromosome condensation rings. ^a^Data from bone marrow, and the remainder from peripheral blood. ^b^Dose estimated by a physics method from the physics group in our hospital.

Bone marrow MSCs derived from 10 unrelated, HLA-mismatched donors were given by intrabone marrow injection or subcutaneous injection into the left hand only on Days 3, 8, 14, 23, 32, 37 and 40, or at Days 28 and 31, respectively, after exposure (Table 2). All procedures for MSC culture were as described previously [12]. Briefly, bone marrow aspirates were collected, mononuclear cells (MNCs) were fractionated with a 1.077 g/ml Ficoll-Paque density gradient and then cultured in Dulbecco's Modified Eagle Medium (DMEM, Gibco, Rockville, MD) supplemented with 10% serum at 37°C in a humidified environment containing 5% CO_2_. After culturing for 24–48 h, non-adherent cells were removed and the adherent layer was further cultured until 70–80% confluence. Then MSCs were harvested and used at passages 3–5. The MSCs harvested were aliquoted and cryopreserved at −80°C in liquid nitrogen including 10% DMSO. When used, the frozen MSCs were thawed fast and melted completely with culture medium at 37°C.

**Table 2.** Infusion of MSCs in the patient after exposure

Frequency	Infusion Time, Day	Doses, Cells/2 ml
1^a^	Day 3	1.1 × 10^7^
2^a^	Day 8	1.55 × 10^7^
3^a^	Day 14	11 × 10^7^
4^a^	Day 23	6.3 × 10^7^
5^a^	Day 32	5.6 × 10^7^
6^b^	Day 28	5.5 × 10^7^
7^b^	Day 31	5.0 × 10^7^
8^a^	Day 37	5.1 × 10^7^
9^a^	Day 40	5.0 × 10^7^

^a^By intrabone marrow injection. ^b^By subcutaneous injection in the skin lesions.

The surface markers of the MSCs were examined by a four-color flow cytometer (EPICSxL-MCL, Beckman Coulter, Fullerton, CA), and the result showed that these cultured cells expressed CD105, CD73, CD90, CD29, CD44 and CD166, but lacked expression of CD45, CD34, CD14, CD79a, HLA-DR, CD31 and CD106 surface molecules. Adipogenic differentiation was assessed by Oil Red O staining, and osteogenic differentiation was analyzed by the von Kossa method. MSCs were also examined for bacteria, fungus and mycoplasma contamination.

**Fig. 1.**
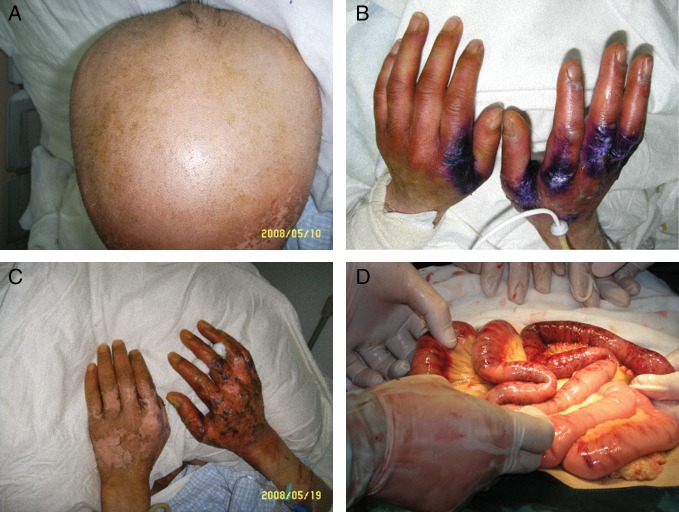
Radiation damage in the patient after exposure. (A) head hair lost; (B) and (C) hands showing erythema and ulceration (days 24 and 44 after exposure, respectively); (D) extensive necrosis of the colon at the surgery.

**Fig. 2.**
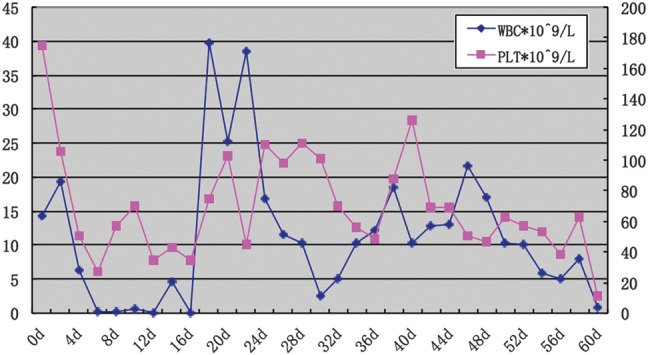
Changes in the WBC and platelet count after exposure.

On Day 11 after exposure, the patient developed alopecia (Fig. 1A), and diarrhea increased gradually to 150 ml per day. On Day 8 after transplantation, the WBC count had increased to 4.68 × 10^9^/l. On Day 12 after transplantation, the WBC and platelet counts had increased to 38.5 × 10^9^/l and 98 × 10^9^/l, respectively (Fig. 2), and diarrhea had increased to 500 ml per day. Bone marrow aspiration showed an active proliferation with tri-lineage hematopoiesis. A stable donor graft was obtained and no rejection occurred. A sustained full donor chimerism (FDC) was observed on Days 12, 28 and 52 after transplantation in the peripheral blood cells and bone marrow cells by short tandem repeat-PCR (STR-PCR), chromosome analysis and HLA-genotyping methods [12].

On Day 16 after transplantation, the lung infection of the patient was cleared, but diarrhea had increased to 500 ml per day, and the skin erythema had progressed to blistering and ulceration (Fig. 1B and C). Treatment with hemostasis, acid suppression agents, Tacrolimus and CD25 monoclonal antibody was employed, but there was no response. On Day 22 after transplantation, the diarrhea had increased to 1200 ml per day and turned to bloody stools with frequent nausea, vomiting and abdominal pain. On Day 36 after transplantation, bloody stools increased to 3000 ml per day, however, the patient's radioactive skin blisters and ulceration on the left hand gradually improved. On Day 40 after transplantation, the bloody stools had further developed to intestinal paralysis, obstruction and shock (blood pressure 50/30 mmHg), although further therapies including high dose hemostasis, pituitrin and stronger support were employed. An emergency surgical operation was conducted and revealed a large amount of bleeding (5000 ml) in the abdominal cavity, complicated with an extensive necrosis and sloughing of the mucosa of the small intestine and the colon (Fig. 1D). A resection of the small bowel and colon was done successfully, in which 4/5 of the small intestine and colon was removed.

On Day 2 after the surgery, the symptoms of diarrhea and bleeding subsided, and other intestinal functions recovered gradually, however, lung infection appeared, associated with high fever. Stronger antibiotics and supportive treatments were given but failed. On Day 12 after surgery, the patient developed sepsis, abscess, shock and disseminated intravascular coagulation. On Day 62 after exposure (Day 14 after the surgery), the patient died of multi-organ failure. The autopsy found degeneration, necrosis and loss of mucosal, epithelial and inherent glands in the whole digestive tract. Furthermore, pulmonary edema with local interstitial fibrosis and multiple fungal infarction foci in the heart and kidneys was also observed.

## DISCUSSION

In the present case, we report on one patient who developed ARS after receiving high-dose radiation in an accident in China. Severe neutropenia, frequent nausea, vomiting, skin ulceration and high fever were observed after exposure, and the radiation dose was estimated to be 14.5 Gy (12.4–17.9 Gy) according to the chromosomes, micronuclei analysis, clinical signs and symptoms [13–16]. The patient was assigned to severity Grade H4 using the METREPOL approach, which suggested irreversible damage to the stem cell pool [3, 17, 18], and because of which the patient received an emergency HLA-mismatched stem cell transplantation combined with continuous infusion of bone marrow MSCs following conditioning with a single dose of fludarabine.

The most important result of these positive findings was that the patient, who received a radiation dose > 10 Gy and was graded H4, achieved a speedy hematopoietic recovery, including recovery of WBC and platelet counts, improvement of his lung infection, and stable full donor engraftment without GVHD. This was in contrast to previous reports from the Chernobyl nuclear accident, the Tokaimura accident in Japan, and the Shanghai accident in China [3, 5, 6, 19]. It should be noted that conditioning with fludarabine and GVHD prophylactic with CSA, MMF and CD25 antibody might have played an important role in ensuring donor engraftment and prevention of GVHD [12, 20, 21]. Alternatively, several studies have shown that MSCs are able to repair injured tissues arising from accidental radiation exposure, hasten hematopoietic recovery, and prevent GVHD when infused simultaneously with MSCs [7–10, 22]. In this case, rapid hematopoietic recovery and healing of skin blisters on the left hand were observed concomitant with the continuous intrabone-marrow and subcutaneous injections of MSCs, suggesting a potential benefit of MSCs in radiation treatments.

Literature has reported that all patients assigned to H4 using the METREPOL approach die within 60 days due to multi-organ failure [3, 17]. In this study, the treatments did not prevent gastrointestinal radiation damage from occurring, which eventually developed into intestinal obstruction, although hematopoietic recovery had been achieved. Although emergency bowel resection successfully released the intestinal obstruction, the patient died on Day 62 from multi-organ failure related to gastrointestinal radiation damage. These results suggested that HLA-mismatched peripheral blood stem cells and MSCs can be used successfully to rescue hematopoietic damage. Drugs or other cells for improvement of other organ damage, such as in the gastrointestinal tract, are urgently needed.

## FUNDING

This work was supported by grants form the “863 Projects” of Ministry of Science and Technology of PR China (No. 2006AA02A109).
